# A Linear Time Solution to the Labeled Robinson–Foulds Distance Problem

**DOI:** 10.1093/sysbio/syac028

**Published:** 2022-04-15

**Authors:** Samuel Briand, Christophe Dessimoz, Nadia El-Mabrouk, Yannis Nevers

**Affiliations:** Département d’informatique et de recherche opérationnelle (DIRO), Université de Montréal, Pavillon André-Aisenstadt, CP 6128 succursale Centre-Ville, Montréal, QC H3C 3J7, Montreal, Canada; Department of Computational Biology, University of Lausanne, Génopode, Quartier UNIL-Sorge - CH-1015, Lausanne, Switzerland; Center for Integrative Genomics, University of Lausanne, Génopode, Quartier UNIL-Sorge - CH-1015, Lausanne, Switzerland; Department of Genetics, Evolution and Environment, University College London, Darwin Building, 99-105 Gower Street, WC1E 6BT, London, UK; Department of Computer Science, University College London, Gower Street, WC1E 6BT, London, UK; SIB Swiss Institute of Bioinformatics, Amphipôle, Quartier UNIL-Sorge - CH-1015, Lausanne, Switzerland; Département d’informatique et de recherche opérationnelle (DIRO), Université de Montréal, Pavillon André-Aisenstadt, CP 6128 succursale Centre-Ville, Montréal, QC H3C 3J7, Montreal, Canada; Department of Computational Biology, University of Lausanne, Génopode, Quartier UNIL-Sorge - CH-1015, Lausanne, Switzerland; Center for Integrative Genomics, University of Lausanne, Génopode, Quartier UNIL-Sorge - CH-1015, Lausanne, Switzerland; SIB Swiss Institute of Bioinformatics, Amphipôle, Quartier UNIL-Sorge - CH-1015, Lausanne, Switzerland

## Abstract

A large variety of pairwise measures of similarity or dissimilarity have been developed for comparing phylogenetic trees, for example, species trees or gene trees. Due to its intuitive definition in terms of tree clades and bipartitions and its computational efficiency, the Robinson–Foulds (RF) distance is the most widely used for trees with unweighted edges and labels restricted to leaves (representing the genetic elements being compared). However, in the case of gene trees, an important information revealing the nature of the homologous relation between gene pairs (orthologs, paralogs, and xenologs) is the type of event associated to each internal node of the tree, typically speciations or duplications, but other types of events may also be considered, such as horizontal gene transfers. This labeling of internal nodes is usually inferred from a gene tree/species tree reconciliation method. Here, we address the problem of comparing such event-labeled trees. The problem differs from the classical problem of comparing uniformly labeled trees (all labels belonging to the same alphabet) that may be done using the Tree Edit Distance (TED) mainly due to the fact that, in our case, two different alphabets are considered for the leaves and internal nodes of the tree, and leaves are not affected by edit operations. We propose an extension of the RF distance to event-labeled trees, based on edit operations comparable to those considered for TED: node insertion, node deletion, and label substitution. We show that this new *Labeled Robinson–Foulds* (LRF) distance can be computed in linear time, in addition of maintaining other desirable properties: being a metric, reducing to RF for trees with no labels on internal nodes and maintaining an intuitive interpretation. The algorithm for computing the LRF distance enables novel analyses on event-label trees such as reconciled gene trees. Here, we use it to study the impact of taxon sampling on labeled gene tree inference and conclude that denser taxon sampling yields trees with better topology but worse labeling. [Algorithms; combinatorics; gene trees; phylogenetics; Robinson–Foulds; tree distance.]

Gene trees, usually derived from gene sequence alignments, represent the phylogenetic relationships between the genes labeling the leaves of the tree. From such representation, we can infer the most plausible scenarios of evolutionary events leading to the observed gene family from an ancestral gene. For this purpose, reconciliation methods (reviewed in Boussau and Scornavacca 2020) embed a given gene tree $T$ into a known species tree $S$. This process results in the labeling of the internal nodes of $T$ with the type of events which gave rise to them. In this article, we address the problem of comparing such *event-labeled trees*, that is, trees with inner nodes labeled with the type of event at the origin of the bifurcation. Note that such an event-labeled tree does not fully represent a “reconciliation” $R$ as defined in the literature, as it should also be indicated for each node of $R$ the position in the species tree where the event took place. However, the event-labeling of $T$ is sufficient to determine the orthology (genes related through speciation), paralogy (genes deriving from a duplication), or xenology (genes related through a horizontal gene transfer) relations between genes, with important functional implications (Gabaldon and Koonin 2013). For example, information on duplication and speciation node labeling is provided for the trees of the Ensembl Compara database (Vilella et al. 2009).

A large variety of pairwise measures of similarity or dissimilarity have been developed for trees with no labels on internal nodes. Among them are the methods based on counting the structural differences between the two trees in terms of path size, bipartitions or quartets for unrooted trees, clades or triplets for rooted trees (Estabrook et al. 1985; Critchlow et al. 1996; Cardona et al. 2010), or those based on minimizing a number of rearrangements that disconnect and reconnect subpieces of a tree such as nearest neighbor interchange (NNI), subtree-pruning-regrafting (SPR), or Tree-Bisection-Reconnection (TBR) moves (Jiang et al. 2000; Allen and Steel 2001; Hickey et al. 2008). While the latter methods are NP-hard (Lin et al. 2012), the former are typically computable in polynomial time. In particular, the Robinson–Foulds (RF) distance, defined in terms of bipartition dissimilarity for unrooted trees and clade dissimilarity for rooted trees (Mittal and Munjal 2015), can be computed in linear (Day 1985), and even sublinear time (Pattengale et al. 2007). For trees with unweighted edges, the RF distance is the most widely used distance, not only in phylogenetics but also in other fields such as in linguistics, for its computational efficiency, intuitive interpretation and the fact that it is a true metric. To address the distance’s drawbacks, such as lack of robustness (a small change in a tree may cause a disproportional change in the distance) and skewed distribution, improved versions of the RF distance have also been developed (Lin et al. 2012; Moon and Eulenstein 2018), among them methods allocating similarity scores between bipartitions rather than simply counting the bipartitions that are different in the two trees (Smith 2020).

Classically defined in terms of bipartition or clade dissimilarity, the RF distance can also be defined in terms of edit operations on tree edges: the minimum number of edge contraction and extension needed to transform one tree into the other (Robinson and Foulds 1981). This formulation is closely related to the Tree Edit Distance (TED) defined on labeled trees (usually rooted, and sometimes with an order on nodes), that is, trees with nodes (including leaves) labeled on the same given alphabet $\Sigma$, arising from many different applications in various fields (parsing, RNA structure comparison, computer vision, genealogical studies, etc). TED (Zhang and Shasha 1989) is defined in terms of a minimum cost path of node deletion (resulting in the deletion of the edge linking this node to its parent), node insertion (resulting in the creation of a new edge), and node change (label substitution). The general version of the problem on unordered labeled trees with a non-constant cost function on edit operations is NP-complete (Zhang et al. 1992), while most restrictions and variants that have been defined for that distance are solvable in polynomial time (Zhang 1993, 1996; Bille 2005; Schwarz et al. 2017).

However, the variants that have been considered for TED do not include the type of event-labeled trees we consider in this article with two different alphabets: an alphabet $\mathcal{L}$ for the leaves corresponding to the genes, and an alphabet $\Lambda$ for the inner nodes corresponding to the events. Moreover, leaves are not affected by edit operations. Notice that the problem is different from that of comparing two reconciliations of a gene tree. Exploring the reconciliation space of a given tree or comparing two different reconciliations, for example, with or without horizontal gene transfers, has been largely addressed in the reconciliation literature (Doyon et al. 2009, 2011; Chan et al. 2015; Huber et al. 2018). Here, we address the problem of comparing different event-labeled trees for the same gene family, in the sense that the trees can differ, not only in labels but also in topology.

We formulate our distance as an extension of the RF distance to inner node-labeled trees. Although the RF distance is not based on biological events, it is the most widely used distance for comparing phylogenetic trees for its intuitive definition in terms of clades or bipartitions of the trees, and its computational efficiency. Therefore, an extension of the RF distance to inner node-labeled trees is not expected to represent true evolutionary events, but should allow capturing the topological and labeling difference between two trees, and should be computable efficiently.

In Briand et al. (2020), we have presented ELRF, a first extension of RF for comparing inner node-labeled gene trees, expressed in terms of trees with a binary node labeling. ELRF is obtained by including a node *flip* operation, alongside edge contractions and extensions. While remaining a metric, ELRF turned out to be much more challenging to compute. As a result, we were only able to propose a heuristic to compute it efficiently.

In this article, we explore a different extension of RF in terms of edit operations on tree nodes rather than on tree edges, which is closer to the TED formulation. We show that, in contrast with ELRF, this new distance is computable in linear time, not only for two but for an arbitrary number of label types. We show that the new distance compares favorably to RF and ELRF by performing simulations on labeled gene trees of 182 leaves. Finally, we use our new distance in the purpose of measuring the impact of taxon sampling on labeled gene tree inference, and conclude that denser taxon sampling yields better predictions at the topological level but leads to worse evolutionary event labeling.

## Notation and Concepts

Let $T$ be a tree with node set $V(T)$ and edge set $E(T)$. Given a node $x$ of $T$, the *degree of $x$* is the number of edges incident to $x$. We denote by $L(T) \subseteq V(T)$ the set of *leaves of $T$*, that is, the set of nodes of $T$ of degree one. Given a set $\mathcal{L}$ (species or genes), a tree $T$ on $\mathcal{L}$ is a tree with a one-to-one relationship between $L(T)$ and $\mathcal{L}$. For simplicity of presentation, in this article, we make no difference between a leaf and the associated element of $\mathcal{L}$.

A node of $V(T) \setminus L(T)$ is called an *internal node*. A tree with a single internal node $x$ is called a *star tree*, and $x$ is called a *star node*. An edge connecting two internal nodes is called an *internal edge*; otherwise, it is a *terminal edge*. Moreover, a *rooted tree* admits a single internal node $r(T)$ considered as the root.

The root is said to be *binary* if it is of degree 2; any other internal node is said to be *binary* if it is of degree 3. The trees considered in this article are such that all internal nodes are of degree at least 3, except the root in the case of rooted trees, which is of degree at least 2.

We call $N(x) = \{y : \{x,y\} \in E(T)\}$ the set of neighbors of an internal node $x$ of $T$.

A *subtree* $S$ of $T$ is a tree such that $V(S) \subseteq V(T)$, $E(S) \subseteq E(T)$ and any edge of $E(S)$ connects two nodes of $V(S)$.

The *bipartition* of an unrooted tree $T$ corresponding to an edge $e=\{x,y\}$ is the unordered pair of clades $L(T_x)$ and $L(T_y),$ where $T_x$ and $T_y$ are the two subtrees rooted, respectively at $x$ and $y$ obtained by removing $e$ from $T$. We denote by ${{\mathcal B}}(T)$ the set of nontrivial bipartitions of $T$, that is, those corresponding to internal edges of $T$.

### The Robinson–Foulds distance (RF)

It is defined for both rooted and unrooted trees. Notice however that computing the RF distance for two rooted trees can be reduced to computing the RF distance for the two corresponding unrooted trees obtained by grafting an edge linking the root to a dummy leaf (Briand et al. 2020). Conversely, the problem of computing the RF distance for two unrooted trees can be reduced to computing it for the rooted trees using an arbitrarily chosen leaf as the root (Day 1985). Notations and theoretical results of this article are given for unrooted trees (notice however that the algorithm presented below is implemented in terms of rooted trees). We denote by ${{\mathcal T}}_{\mathcal{L}}$ the set of unrooted trees on $\mathcal{L}$. Given two unrooted trees $T$ and $T'$ of ${{\mathcal T}}_{\mathcal{L}}$, the Robinson–Foulds (RF) distance between $T$ and $T'$ is the size of the symmetric difference between the bipartitions of the two trees. More precisely,

$$
\[{\rm RF}(T,T') = |{{\mathcal B}}(T) \setminus {{\mathcal B}}(T')| + |{{\mathcal B}}(T') \setminus{{\mathcal B}}(T)|.\]
$$

The RF distance is equivalently defined in terms of an edit distance on edges (Robinson and Foulds 1981). However, as for node-labeled trees an additional substitution operation on node labels will be required, for the sake of standardization, we reformulate the edit operations to operate on nodes rather than on edges.

Definition 1 (Node edit operations). *Two edit operations on the nodes of a tree $T$ are defined as follows:*

- **Node deletion ($Del$):** *Let $x$ be an internal node of $T$ which is not a star node and $y$ be a neighbor of $x$ which is not a leaf. Deleting $x$ with respect to $y$ means making the neighbors of $x$ become neighbors of $y$. This is equivalent to deleting $y$ with respect to $x$ and can be seen as contracting the edge $\{x,y\}$. More precisely, $Del(T,x,y)$ is an operation transforming the tree $T$ into the tree $T'$ obtained from $T$ by removing the edge $\{x,z\}$ for each $z \in N(x)$, creating the edge $\{y,z\}$ for each $z \in N(x)\setminus \{y\}$, and then removing node $x$.*
- **Node insertion ($Ins$):** *Let $y$ be an internal node of $V(T)$ of degree at least 4. Inserting $x$ as a neighbor of $y$ entails making $x$ the neighbor of a subset $Z \subsetneq N(y)$ such that $|Z| \geq 2$. This can be seen as an edge extension operation creating a new edge $\{x,y\}$. More precisely, $Ins(T,x,y,Z)$ is an operation transforming the tree $T$ into the tree $T'$ obtained from $T$ by removing the edges $\{y,z_i\}$, for all $z_i \in Z$, creating a node $x$ and a new edge $e = \{x,y\}$, and creating new edges $\{x, z_i\}$, for all $z_i \in Z$.*

Now, the *Robinson–Foulds* distance $RF (T,T')$ between $T$ and $T'$ is the size of a shortest path of edge edit operations (i.e., node insertion and node deletion) transforming $T$ into $T'$.

Call a *bad edge* of $T$ with respect to $T'$ (or similarly of $T'$ with respect to $T$; if there is no ambiguity, we will omit the “with respect to” precision) an edge representing bipartitions which are not shared by the two trees, that is, an edge of $T$ defining a bipartition of ${{\mathcal B}}(T)$ which is not in ${{\mathcal B}}(T')$. An edge which is not bad is said to be *good*. Terminal edges are always good.

## Generalizing the Robinson–Foulds Distance to Labeled Trees

We are interested in comparing trees with information on internal nodes, which is the label of interest in this article. Therefore, in this article, we say that a tree $T$ is *labeled* if each internal node $x$ of $T$ has a label $\lambda(x) \in \Lambda$, $\Lambda$ being a finite set of labels. We denote by ${{\mathcal T}}_{\mathcal{L,\Lambda}}$ the set of unrooted and labeled trees on $\mathcal{L}$ with labels from $\Lambda$. For gene trees, labels are reasonably the type of event leading to the bifurcation, typically duplications, speciations, or horizontal gene transfers, but may also represent the mapping to the nodes of the species tree. Moreover, we mean by an “unlabeled tree” a tree with no labels on internal nodes (but yet with the labels $\mathcal{L}$ on leaves).

**Figure 1.**
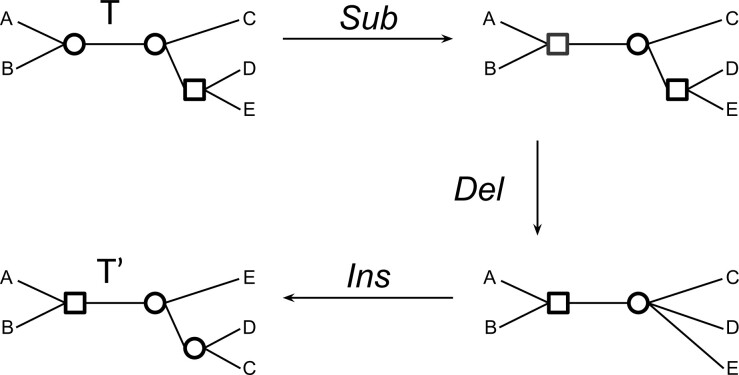
The transformation of a tree $T$ into a tree $T'$ depicting the three edit operations on nodes. From top to bottom: node label substitution (the neighbor of $A$ and $B$, circle to square), node deletion (the neighbor of $D$ and $E$), and node insertion (the neighbor of $D$ and $C$).

We generalize the RF distance to labeled trees by generalizing the edit operations defined above. This is simply done by introducing a third operation for node labels editing (Fig. 1).

Definition 2 (Labeled node edit operations). *Three edit operations on internal nodes of a labeled tree $T$ are defined as follows:*

- **Node deletion:** *$Del(T,x,y)$ is an operation deleting an internal node $x$ of $T$ with respect to a neighbor $y$ of $x$ which is not a leaf, defined as in Definition 1.*
- **Node insertion:** *$Ins(T,x,y,Z,\lambda)$ is an operation inserting an internal node $x$ as a new neighbor of a nonbinary node $y$, and moving $Z \subsetneq N(y)$ such that $|Z| \geq 2$, to be the neighbors of $x$, as defined in Definition 1. In addition, the inserted node $x$ receives a label $\lambda \in \Lambda$.*
- **Node label substitution:** *$Sub(T,x,\lambda)$ is an operation substituting the label of the internal node $x$ of $T$ with $\lambda \in \Lambda$.*

For two trees $T$, $T'$ of ${{\mathcal T}}_{\mathcal{L,\Lambda}}$, we call the *Labeled Robinson–Foulds* distance between $T$ and $T'$ and denote by ${\rm LRF}(T,T')$ the size of a shortest path of labeled node edit operations transforming $T$ into $T'$. The two following lemmas state that, similarly to RF, LRF is a true metric. Moreover, LRF is exactly RF for unlabeled trees.

In the following, the *unlabeled version of* a tree $T\in{{\mathcal T}}_{\mathcal{L,\Lambda}}$ is simply $T$ ignoring its node labels.

Lemma 1. *The function* ${\rm LRF}(T,T')$*assigning to each pair $(T,T')\in {{\mathcal T}}_{\mathcal{L,\Lambda}}^2$ the size of a shortest path of node edit operations transforming $T$ into $T'$ defines a distance on ${{\mathcal T}}_{\mathcal{L,\Lambda}}$.*

Proof for this lemma is available in the Appendix.

The next lemma directly follows from the fact that node substitutions are never applied in case of a label set $\Lambda$ restricted to a single label (i.e., for unlabeled trees).

Lemma 2. *If $\Lambda$ is restricted to a single label, then for each pair $(T,T')\in {{\mathcal T}}_{\mathcal{L,\Lambda}}^2$, ${\rm LRF}(T,T') = {\rm RF}(T,T')$.*

A previous extension of RF to labeled trees, based on edit operations on edges rather than on nodes, was introduced in Briand et al. (2020). This distance, which we call ELRF, was defined on three operations:

- Edge extension $Ext(T,x,X)$ creating an edge $\{x,y\}$ and defined as a node insertion ${\rm Ins}(T,y,x,X,\lambda(x))$ inserting a node $y$ as a neighbor of $x$ and assigning to $y$ the label of $x$;
- Edge contraction ${\rm Cont}(T,\{x,y\})$ is equal to a node deletion ${\rm Del}(T,y,x)$ deleting $y$, but requires that $\lambda(x) = \lambda(y)$, a condition which is not requested for LRF;
- Node flip ${\rm Flip}(T,x,\lambda)$ assigning the label $\lambda$ to $x$. We use the term “flip” to emphasize the fact that ELRF only supports two kinds of labels, unlike LRF.

Given two labeled trees $T$ and $T'$ of ${{\mathcal T}}_{\mathcal{L,\Lambda}}$, ${\rm ELRF}(T,T')$ is the size of the shortest path of edge extension, edge contraction, and label flip required to transform $T$ to $T'$. The following lemma makes the link between LRF and ELRF.

Lemma 3. *For any pair $(T,T')\in {{\mathcal T}}_{\mathcal{L,\Lambda}}^2$,*

$$
\[{\rm LRF}(T,T') \leq {\rm ELRF}(T,T').\]$$

The proof for this lemma is available in the Appendix.

We now turn our attention to computing the edit distance ${\rm LRF}(T,T')$ for a pair $(T, T')$ of trees of ${{\mathcal T}}_{\mathcal{L,\Lambda}}$.

### Reduction to Islands

We define a subdivision of the two trees into pairs of maximal subtrees that can be treated separately.

While a good edge $e$ in $T$ has a corresponding good edge $e'$ in $T'$ (the one defining the same bipartition), a bad edge in $T$ has no corresponding edge in $T'$. However, those bad edges may be grouped into pairs of corresponding *islands* (called maximum bad subtrees in Briand et al. (2020)), as defined below.

Definition 3 (Islands). *An *island* of $T$ is a maximal subtree $I$ such that all its internal edges are bad edges of $T$, and all its terminal edges are good edges of $T$. The *size* of $I$, denoted $\epsilon(I)$, is its number of internal edges.*

Notice that an island $I$ of $T$ may have no internal edge at all, i.e. it may be restricted to a star tree (if $\epsilon(I)=0$). Notice also that each bad edge of $T$ belongs to a single island, while each good edge belongs to exactly two islands of $T$ if it is an internal edge of $T$, or to a single island if it is a terminal edge of $T$.

The following lemma (similar to Lemma 3 in Briand et al. (2020)) shows that there is a one-to-one correspondence between the islands of $T$ and those of $T'$.

Lemma 4. *Let $I$ be an island of $T$ with the set $\{e_i\}_{1 \leq i \leq k}$ of $I$-terminal edges, and let $\{e'_i\}_{1 \leq i \leq k}$ be the corresponding set of edges in $T'$. Then the subtree $I'$ of $T'$, containing all $e'_i$ edges as $I'$-terminal, is unique. Moreover, it is an island of $T'$.*

The proof for this lemma is available in the Appendix.

**Figure 3.**
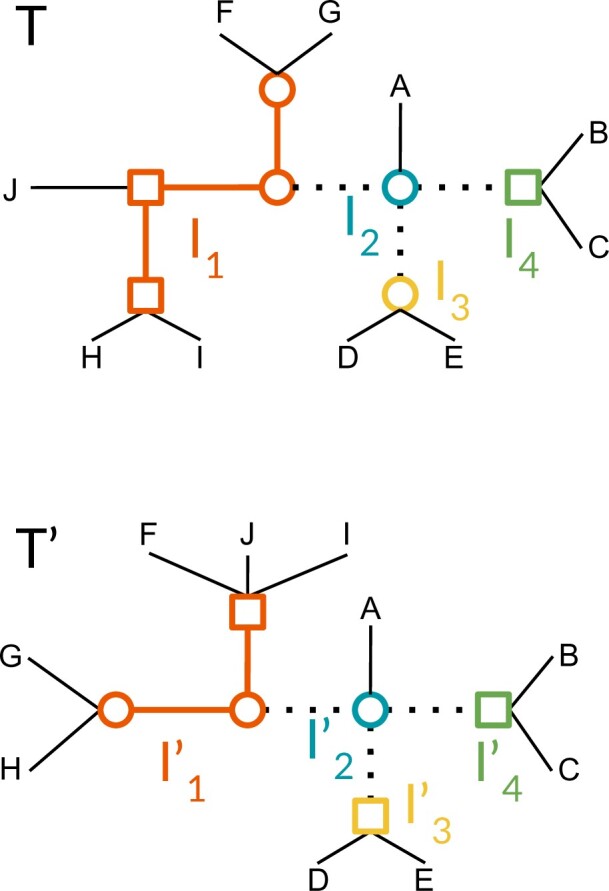
Two trees $T$ and $T'$ on ${{\mathcal T}}_{\mathcal{L,\Lambda}}$ for $\mathcal{L}= \{A,B,C,D,E,F,I,J\}$, with a binary labeling of internal nodes (squares and circles). Dotted lines represent good internal edges, solid lines represent bad edges, and thin lines represent terminal edges (which are good edges). This representation highlights the subdivision of the two trees into the island pairs ${{\mathcal I}}_{(T,T')}= \{(I_1,I'_1), (I_2,I'_2), (I_3,I'_3), (I_4,I'_4)\}$. Notice that each dotted line is a terminal edge of its two adjacent islands.

For any island $I$ of $T$, let $I'$ be the corresponding island of $T'$. We call $(I,I')$ an *island pair* of $(T,T')$ (Fig. 3).

Now, let ${{\mathcal I}}_{(T,T')} = \{(I_1,I'_1), (I_2,I'_2), \ldots, (I_n,I'_n)\}$ be the set of island pairs of $(T,T')$. For $1 \leq i \leq n$, let ${{\mathcal P}}_i$ be a shortest path of labeled node edit operations transforming $I_i$ into $I'_i$. Then the path ${{\mathcal P}}$ obtained by performing consecutively ${{\mathcal P}}_1, {{\mathcal P}}_2, \ldots, {{\mathcal P}}_n$ (that we represent later as ${{\mathcal P}}_1. {{\mathcal P}}_2. \ldots. {{\mathcal P}}_n$) clearly transforms $T$ into $T'$. Therefore we have

$$
\[{\rm LRF}(T,T') \leq \sum_{i=1}^{n} {\rm LRF}(I_i, I'_i). \]$$

As described in Briand et al. (2020), one major issue with ELRF is that good edge contractions may not be avoided in a shortest path of edit operations transforming $T$ into $T'$, resulting in island merging. In other words, treating island pairs separately may not result in an optimal scenario of edit operations under ELRF, preventing the above inequality from being an equality. Interestingly, the equality holds for the LRF distance, as we show in the next section.

### Computing the LRF Distance on Islands

We require an additional definition. Two trees $I$ and $I'$ of an island pair are said to *share a common label* $l \in \Lambda$ if there exist $x \in V(I)$ and $x'\in V(I')$ such that $\lambda (x) = \lambda (x') =l$. If $I$ and $I'$ do not share any common label, then $(I,I')$ is called a *label-disjoint* island pair.

**Figure 4.**
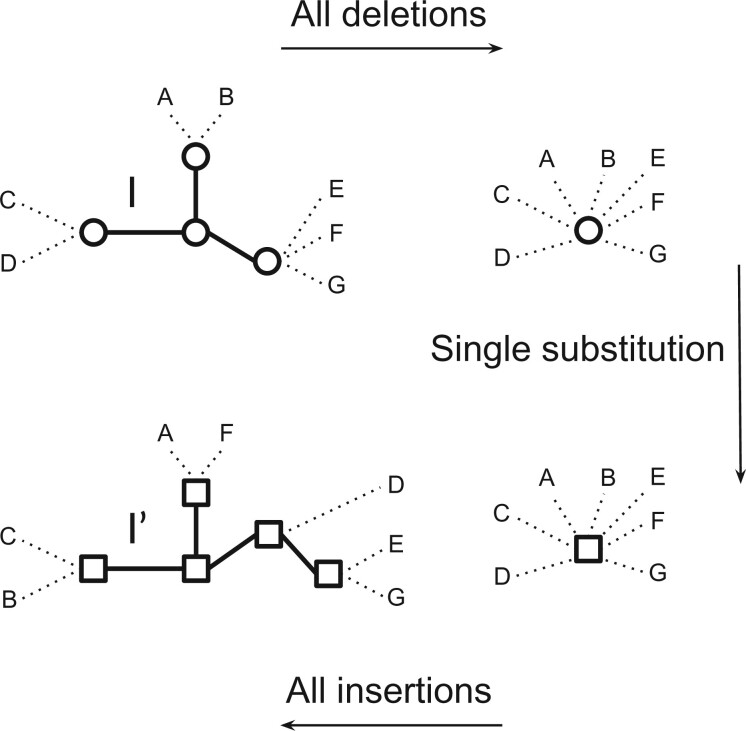
An optimal sequence of edit operations for the island pair $(I,I')$.

Now let $(I,I')$ be an island pair. Transforming $I$ into $I'$ can be done by reducing $I$ into a star tree by performing a sequence of node deletions (if any, i.e., if $I$ is not already a star tree) and then raising the star tree by inserting the required nodes to reach $I'$. Only the unique node not deleted during the first step might require a label substitution; for all inserted nodes, the label can be chosen to match that of $I'$. However, if $I$ and $I'$ share a common label $l$ among their internal nodes, then the deletions can be done in a way such that the surviving node $x$ of $I$ is one with label $\lambda(x) = l$, thus avoiding the need for any substitution. The number of required operations is thus $\epsilon(I)$ deletions, followed by zero or one substitution, followed by $\epsilon(I')$ insertions. Alternatively, the problem can be seen as one of reducing the two trees into star trees by performing $\epsilon(I)+\epsilon(I')$ deletions, in a way reducing the two islands into two star trees sharing the same label, if possible. Figure 4 depicts an example of such tree editing for a label-disjoint island pair.

The following lemma shows that reducing islands to star trees is optimal.

Lemma 5. *Let $(I,I')$ be an element of ${{\mathcal I}}_{(T,T')}$. Then:*

- *If $I$ and $I'$ share a common label, then $LRF(I,I') = \epsilon(I) + \epsilon(I')$.*
- *Otherwise $LRF(I,I') = \epsilon(I) + \epsilon(I') + 1$.*

The proof for this lemma is available in the Appendix.

We have obviously ${\rm LRF}(T,T') \leq \sum_{(I,I') \in {{\mathcal I}}_{(T,T')}} {\rm LRF}(I,I')$. It remains to show that the symmetrical inequality also holds, that is, we cannot do better by merging islands, and thus pairs of islands can be considered separately.

The following lemma states that we can always find a sequence of operations, at each step maintaining or increasing the number of islands, that is, never merging islands. For a path ${{\mathcal P}}=(o_1, o_2, \ldots o_p)$ transforming a tree $T$ into a tree $T'$ and $1 \leq k \leq p$, denote by $T_k$ the tree obtained from $T$ after performing the sub-sequence of operations ${{\mathcal P}}_k=(o_1, \ldots o_k)$.

Lemma 6. *Let $T$ and $T'$ be two trees of ${{\mathcal T}}_{\mathcal{L,\Lambda}}$. There is a shortest path ${{\mathcal P}}=(o_1, o_2, \ldots o_p)$ of edit operations transforming $T$ into $T'$ such that for each $k$, $2\leq k \leq p$, $|{{\mathcal I}}(T_{k-1},T')| \leq |{{\mathcal I}}(T_{k},T')|$.*

The proof for this lemma is available in the Appendix.

**Figure 5.**
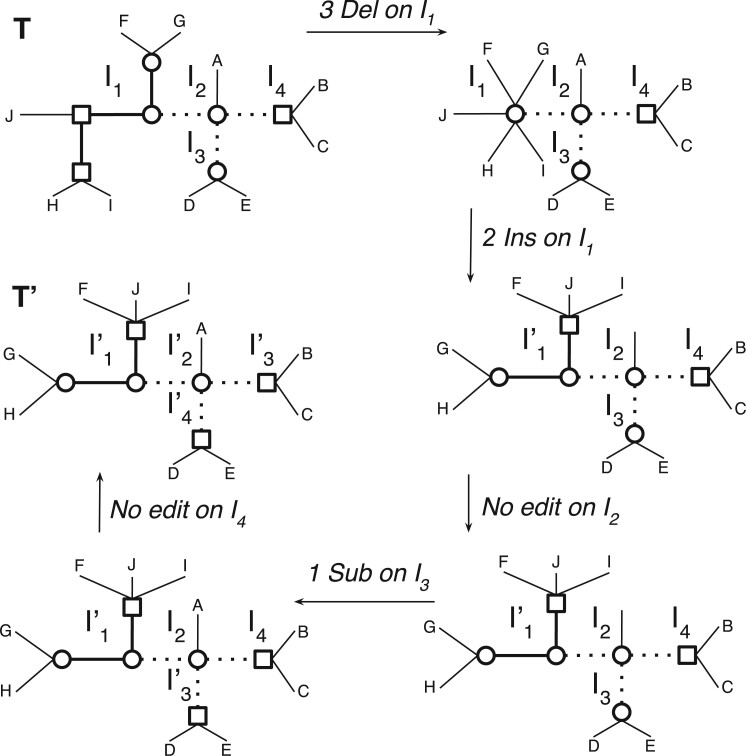
A path ${{\mathcal P}}$ transforming $T$ into $T'$ of the form ${{\mathcal P}}_1. {{\mathcal P}}_2. {{\mathcal P}}_3. {{\mathcal P}}_4$, each ${{\mathcal P}}_i$ being a shortest path for the island pair $(I_i, I'_i)$. Here $|{{\mathcal P}}_1|=6$, $|{{\mathcal P}}_2|=0$, $|{{\mathcal P}}_3|=1$, and $|{{\mathcal P}}_4|=0$.

We are now ready to prove the equality leading to the efficient computation of the LRF distance of two trees (see Fig. 5 for an example).

Theorem 1. *Let ${{\mathcal I}}_{(T,T')} = \{(I_1,I'_1), (I_2,I'_2), \cdots, (I_n,I'_n)\}$ be the island pairs of $T$ and $T'$. Then*

$$
\[LRF(T,T') = \sum_{i=1}^{n} LRF(I_i, I'_i)\]$$

The proof for this theorem is available in the Appendix.

The next result directly follows from Lemma 5 and Theorem 1.

Corollary 1. *Let ${{\mathcal I}}_{(T,T')} = \{(I_1, I'_1), (I_2, I'_2), \cdots, (I_n,I'_n)\}$ be the island pairs of $T$ and $T'$ and $\delta$ be the number of label-disjoint pairs. Then*

$$
\[{\rm LRF}(T,T') = \sum_{i=1}^{n}(\epsilon(I_i)+\epsilon(I'_i)) \: + \: \delta.\]$$

## Algorithm

We present our algorithm for computing the LRF distance (Algorithm 1). The input is a pair of trees $T_1$, $T_2$ of ${{\mathcal T}}_{\mathcal{L,\Lambda}}$. We show that ${\rm LRF}(T_1,T_2)$ can be computed in time $\mathcal{O}(n)$, where $n=|\mathcal{L}|$.

The algorithm operates on rooted trees, without loss of generality. Indeed, recall that we can define a bijection turning an unrooted tree over $n$ leaves into its corresponding rooted tree using an arbitrary leaf as a root (see Notation and Concepts section). Note also that there is a bijection between the bipartitions defined for each branch of the unrooted tree and the clades defined by each branch of its rooted counterpart.

The key to devising an efficient algorithm is to index the clades of $T_1$ in $\mathcal{O}(1)$. This can be achieved using the data structure of Day (1985), who used it to introduce an algorithm to compute the conventional (unlabeled) Robinson–Foulds distance in $\mathcal{O}(n)$. To efficiently index the clades of $T_1$, Day renumbers the leaves of $T_1$ using a postorder sequence. With this representation, each clade is defined by a contiguous sequence of leaves, for example, $[5, 6, 7, 8]$, which can thus be summarized by its smallest and highest values $[5, 8]$. He stores the clades in a table $X$ of $n$ rows, where each clade $[l,r]$ is stored either in row $l$ or $r$. In this way, we can use $X$ to check for the existence of clade $[l,r]$ in constant time.

**Figure 6.**
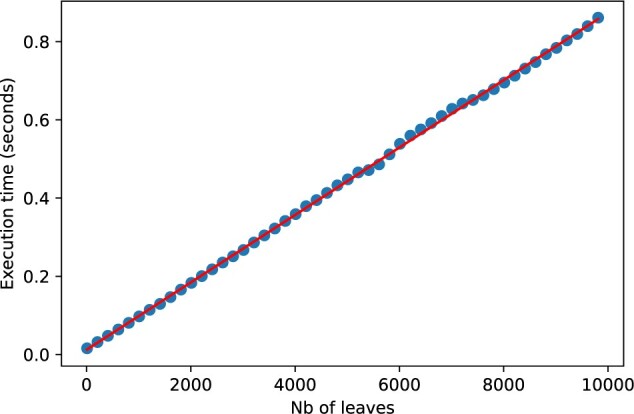
Our implementation of LRF in Python exhibits a linear runtime on random trees of up to 10,000 leaves. Even on these large trees, it takes less than a second to compute LRF on an AMD EPYC 7272 processor.

Our algorithm LRF (Algorithm 1) consists of four tree traversals, followed by one traversal of the aforementioned table $X$. The first tree traversal, of $T_1$, renumbers the leaves of $T_1$ in postorder and stores the clades in $X$ (line 1, $buildX$). The second tree traversal, of $T_2$, identifies the clades of $T_2$ that are shared with $T_1$ using the efficient $X$ lookup structure and marks the “good” clades (corresponding to good edges) as such in $X$ (line 2, $findGoodClades$). The third and fourth tree traversals, of $T_1$ and $T_2$, respectively (lines 3 and 4, $getIslands$), identify the islands that are separated by the good edges recorded in $X$, and update $X$ with the size and labels present in the islands of $T_1$ and $T_2$, respectively. Finally, we traverse $X$ and use the size and labels of all matching islands to compute the LRF edit distance, using the formulae of Appendix- Corollary 1 (lines 8–9). Note that for a fixed number of labels, testing for the presence of a common label can be done in a constant time with respect to $n$. We provide more details on the implementation, pseudocode and complexity of the tree traversal in the Appendix. We provide an open source implementation of LRF in Python as part of the pyLabeledRF package (https://github.com/DessimozLab/pylabeledrf). To empirically confirm the linearity of the algorithm, we computed the LRF distance between random trees of size up to 10,000 leaves. The run time averaged over 100 trees per point was almost perfectly linear (Fig. 6).

## Experimental Results

To illustrate the usefulness of LRF, we performed two experiments. First, we compared LRF with RF and ELRF on a labeled gene tree with random edits. Second, we used LRF to tackle an open question in orthology inference: does labeled gene tree inference benefit from denser taxon sampling?

### Empirical Comparison of LRF with RF and ELRF

To get a first sense of LRF’s ability to measure the actual number of edits between two trees, we performed a simulation study alongside RF and ELRF. We retrieved the labeled tree associated with human gene NOX4 from Ensembl release 99 (Yates et al. 2020), containing 182 genes, including speciation and duplication nodes. Next, we introduced a varying number of random edits, with 10 replicates, as follows: with probability 0.3, the label of one random internal node was substituted (from a speciation label into a duplication one or vice versa); the remaining probability of 0.7 was evenly distributed among all internal edges (each implying a potential node deletion) and all nodes of degree $>3$ (each providing the opportunity of a potential node insertion). For ELRF, consistent with its underlying model, we added the requirement that edge removal only affects edges with adjacent nodes with the same label.

**Figure 7.**
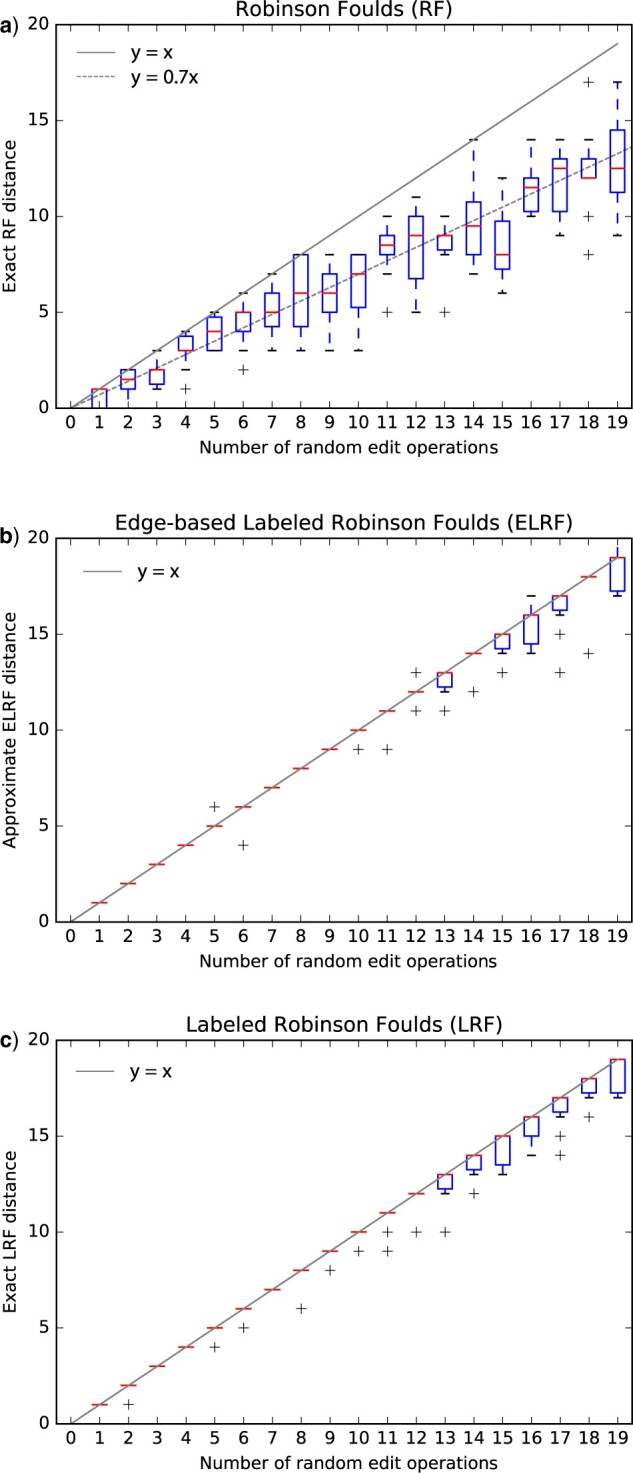
Empirical comparisons of the distance inferred for an increasing number of random edit operations (node insertion, deletion, and substitution) on the NOX4 gene tree (182 leaves), using the classical RF distance (top), the ELRF approximation (Briand et al. 2020; middle), and the LRF exact distance (bottom).

For each of RF, LRF, and ELRF, we provide the distance as a function of the number of random edits (Fig. 7). As expected, the conventional RF distance returns the smallest values because it ignores labels; it however tracks quite well the expected number of node insertion and/or removal (dashed line). The two labeled RF alternatives performed similarly, but the heuristic for ELRF occasionally exceeded the true number of edit operations—a shortcoming that we do not have with LRF, as we have an exact algorithm for this distance. Both labeled RF variants tracked better the actual number of changes, until around 13 edits for LRF or ELRF, after which the minimum edit path starts to be often shorter than the actual sequence of random edits.

### The Effect of Denser Taxon Sampling on Labeled Gene Tree Inference

We used LRF to assess the effect of species sampling for the purpose of labeled gene tree reconstruction. Consider the problem of reconstructing a labeled tree corresponding to homologous genes from 10 species. Our question is: is it better to infer and label the tree using these 10 species alone, or is it better to use more species to infer and label the tree, and then prune the resulting tree to only contain the leaves corresponding to the original 10 species? In principle, denser sampling allow to account for more information when resolving the relationship between genes, and it is known to improve unlabeled phylogenetic inference (Nabhan and Sarkar 2011). It is unclear however how the added species affects the labeling, in one hand, improved sampling should make easier to solve difficult case like hidden paralogy (a duplication node appearing as a speciation node). In the other hand, an increased number of internal nodes may make the labeling more sensitive to errors in the topology.

First, using ALF (Dalquen et al. 2012), we simulated the evolution of the genomes of 100 extant species from a common ancestor genome containing 100 genes (*Parameters*: root genome with 100 genes of 432 nucleic acids each; species tree sampled from a birth–death model with default parameters; sequences evolved using the WAG model, with Zipfian gap distribution; duplication and loss events rate of 0.001). In the simulation, genes can mutate, be duplicated or lost. All the genes in the extant species can thus be traced back to one of these 100 ancestral genes and be assigned to the corresponding gene family. The 100 true gene trees, including speciation and duplication labels, are known from the simulation. In our run, the resulting gene trees had in average 100.11 leaves with a minimum of 65 and a maximum of 156.

To evaluate the inference process, among the 100 species, we randomly selected nested groups of 10, 20, 30, 40, 50, 60, 70, 80, and 90 species. We considered the 10 species in the first group as the species of interest. All other species were used to potentially improve the reconstruction of the gene trees for the first 10 genomes. Then, for each group, we aligned the protein sequences translated from homologous genes using MAFFT L-INS-i (Katoh and Standley 2013), inferred phylogenetic trees from the alignments using FastTree (Price et al. 2010), and annotated their nodes using either the species tree reconciliation or the species overlap algorithm (Van der Heijden et al., 2007) as implemented in the ETE3 python library (Huerta-Cepas et al., 2016). Thus, the nodes of the tree were labeled as either duplication or speciation. Finally, we pruned both the inferred gene trees and the true trees to include only genes corresponding to the 10 species of interest. The resulting trees had in average 10.27 leaves. However, in our run, one pruned tree ended up containing only two genes (due to losses on early branches) and was thus excluded from the rest of the analysis. In order to assess the influence of the accessory species on the node labeling, we used a variant of the same strategy where the tree annotation step and pruning step were reversed, and the labeling of the simulated tree was only done based on the pruned tree with the 10 species of interest.

**Figure 8.**
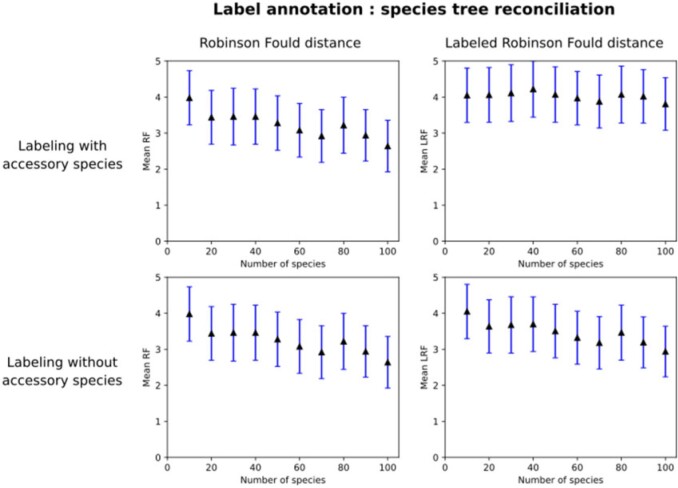
Denser taxon sampling decreases labeled tree estimation error: labeled gene trees reconstructed with an increasing number of auxiliary genomes (i.e., obtained by including the additional genomes during tree inference and labeling, followed by pruning) have a smaller RF (left column) and LRF (right column) distance to the true trees under different annotation strategy. Trees are labeled using the species tree reconciliation algorithm with (top row) or without (bottom row) the accessory species. The decrease of LRF is less important when these species are also used for labeling by species tree reconciliation methods (upper right) than when they are removed prior to reconciliation (lower right). Error bars depict 95$\%$ confidence intervals around the mean.

We used RF and LRF to assess the distance between the estimated and true labeled trees, for the various numbers of auxiliary genomes considered. For each number of accessory species and over all annotation scenarios, we computed the mean RF and LRF distance over all gene trees (Fig. 8, Fig. S1 of the Supplementary material available on Dryad at http://dx.doi.org/10.5061/dryad.2bvq83bpr).

The mean error either reflects the topological error (measured by the RF distance) or both topological and labeling errors (LRF distance). As the RF distance shows, topological error decreases as the number of auxiliary species increases. By contrast, the total error as measured by LRF does not substantially decrease when species reconciliation is performed on the augmented data set (i.e., including accessory species; Fig. 8 upper right). This is due to an increase in labeling errors when the auxiliary species are included, which counterbalances the reduction in topological errors observed on the augmented trees. Thus, the best performance (lowest topological *and* labeling error) is obtained when we infer the tree using the augmented data set but perform the reconciliation after the additional species are removed (Fig. 8 lower right).

If we use the alternative approach of “species overlap” to infer the speciation and duplication nodes (Van der Heijden et al. 2007), we also observe this effect, albeit to a much lesser extent (Fig. S1 of the Supplementary material available on Dryad).

In conclusion, this simulation study indicates that denser species sampling generally improves gene tree inference but, if kept during reconciliation, these accessory species induce a higher event-labeling error if the true augmented species tree is provided as input. The best results are obtained by performing tree inference on the augmented data but reconciliation on the pruned trees. The improvement on the topological inference of the gene tree is in line with our expectations, confirming that increased sampling is generally beneficial even to solve relations between a finite number of genes and should be preferred whenever tractable. It is possible than a reduced “smart” sampling optimizing for diversity and to add resolution to the longest branches would be enough to reproduce most of benefit of a higher sampling. Accordingly, the higher topological improvement gained from accessory species is observed when adding the first 10 species. The observed effect of higher sampling on labeling gene trees is likely due to the sensitivity to errors of the species tree reconciliation algorithm. Indeed, even if the topology of the “target” species tree is improved by denser sampling, the absolute number of topological error on the larger tree is higher and single topological error can lead to drastic changes (principally overannotation of duplication events). The potential benefit of using higher sampling for labeling (e.g., case of hidden paralogy when the topology of the smallest gene tree is not enough for accurate labeling) seems to not be common enough in our simulation to offset this effect.

## Conclusion

The LRF distance introduced here overcomes the major drawback of ELRF, namely the lack of an exact polynomial-time algorithm. Indeed, with ELRF, minimal edit paths can require contracting “good” edges, that is, edges present in the two trees (Briand et al. 2020). By contrast, with LRF, we demonstrated that there is always a minimal path which does not contract good edges. Better yet, we proved that $LRF$ can be computed exactly in linear time. The new formulation also maintains other desirable properties: being a metric, even for an arbitrary number of label types, and reducing to the conventional RF distance in the presence of trees with only one type of label.

The LRF distance is an extension of the RF distance, which is defined with a unit weight for the two edit operations, as otherwise there would be no correspondence with the definition using bipartitions and clades. Moreover, as edge or node insertion and deletion have no biological, but rather topological, meaning, it is hard to see on which criteria weights for these operations would be assigned. In the case of LRF however, changing a node label is related to an evolutionary event, and some substitutions from a duplication to a speciation node lead to an impossible evolutionary scenario. Banning those substitutions implies strong constraints on the intermediate allowed topologies. Alternatively, we may be interested in giving different weights to insertion/deletion versus substitution operations, penalizing node substitution more than node insertion/deletion. In that case however, our algorithm would not be exact anymore as merging islands may result in fewer substitution events. Accounting for the feasibility of substitution events or accounting for weighted events are interesting avenues for future work.

Finally, LRF constitutes a clear improvement over RF in the context of gene tree benchmarking, where trees inferred by various reconciliation models are compared using a distance measure (Altenhoff et al. 2016; Morel et al. 2020). Such an application was illustrated in the simulation study of the previous section, in which we observed that denser taxon sampling improves gene tree inference at the topological level but that it worsens tree reconciliation. This latter result could not have been obtained solely using RF, which serves to illustrate the biological relevance of LRF.

## Figures and Tables

**Figure 2.**
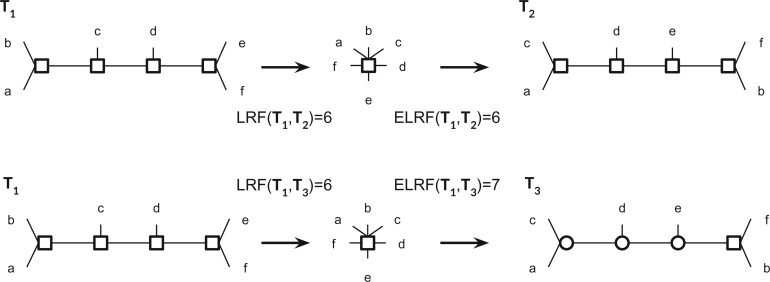
The transformation of a tree $T_1$ into a tree $T_2$ (respectively $T_3$) depicting where the equality (respectively the inequality) is strict between ${\rm LRF}(T_1,T_2)$ and ${\rm ELRF}(T_1,T_2)$ (respectively ${\rm LRF}(T_1,T_3)$ and ${\rm ELRF}(T_1,T_3)$).

**Algorithm 1:**
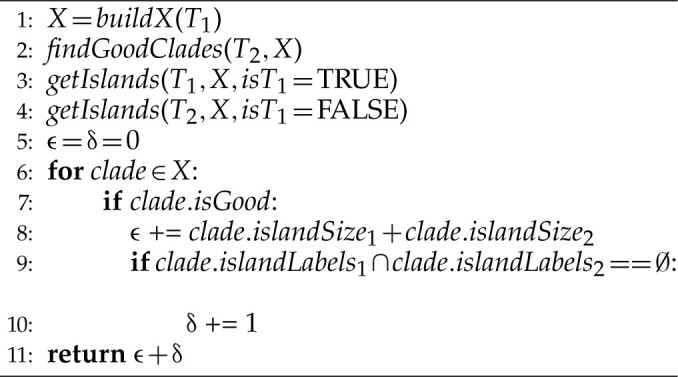
LRF($T_1,T_2$)

**Algorithm 2:**
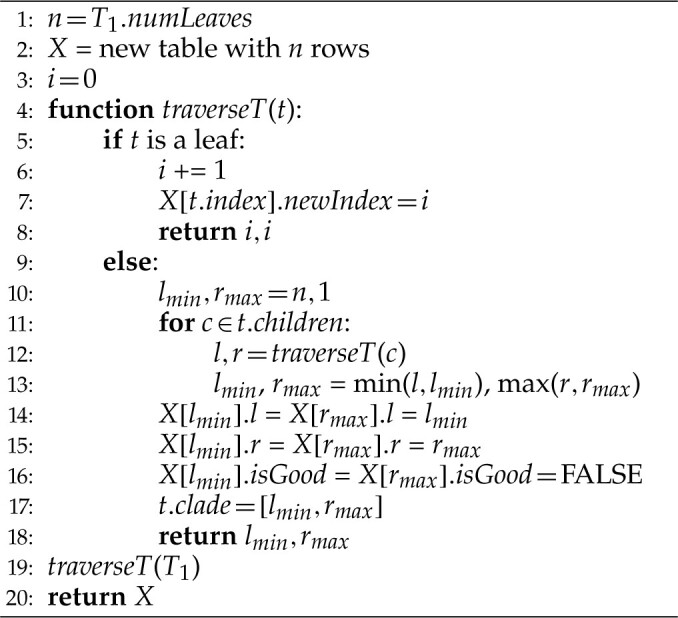
buildX($T_1$)

**Algorithm 3:**
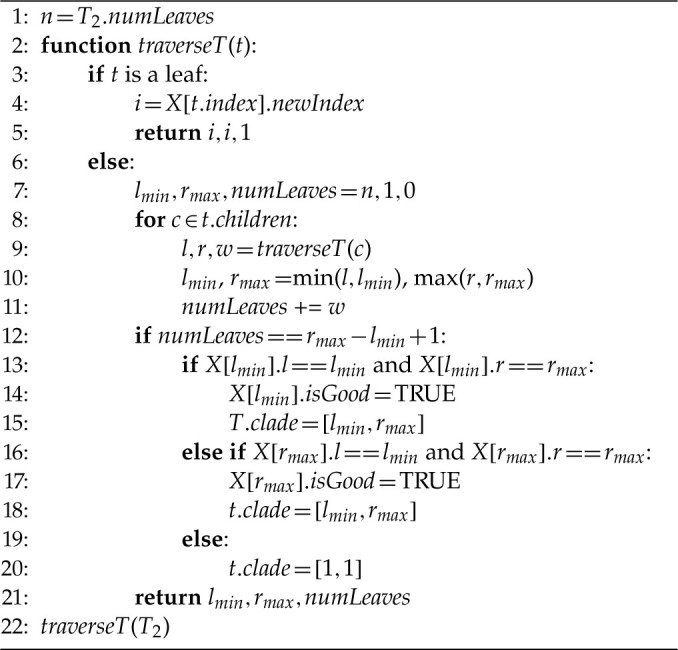
findGoodClades($T_2,X$)

**Algorithm 4:**
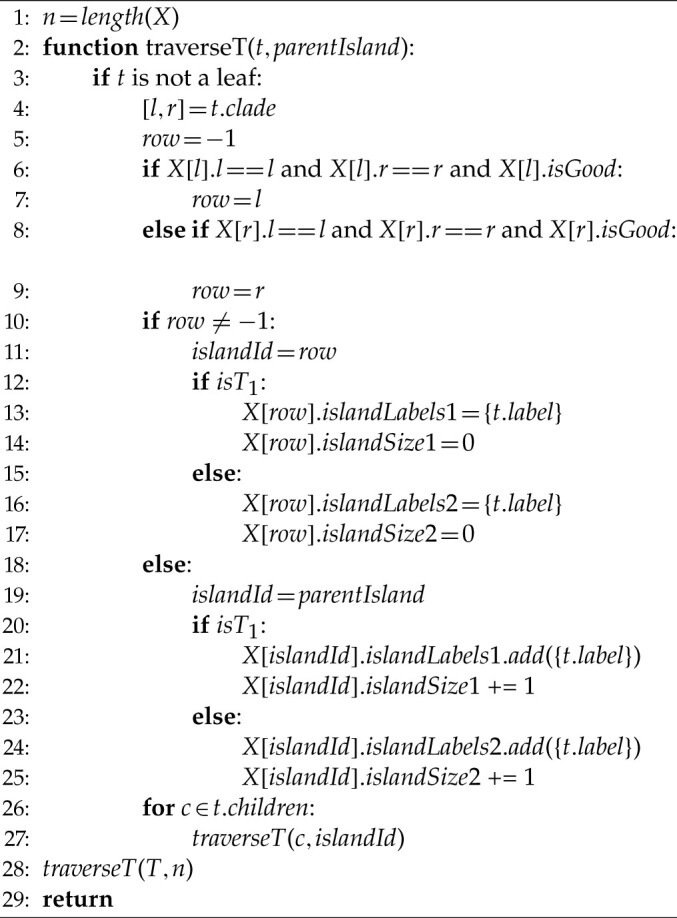
getIslands($T, X, isT_1$)

## Data Availability

Data and scripts for the experimental analyses are available online at Dryad at the URL provided as a supplementary file. The software written in Python is available in the $\texttt{pylabeledrf}$ repository at https://github.com/DessimozLab/pylabeledrf.

## Supplementary material

Data available from the Dryad Digital Repository: http://dx.doi.org/10.5061/dryad.2bvq83bpr.

## APPENDIX

### Proofs of Lemmas and Theorems

**Proof of Lemma 1**

Proof.
The non-negative, identity, and triangular inequality conditions are obvious. For the symmetric condition, notice that we can reverse every edit operation in a path from $T$ to $T'$ to obtain a path from $T'$ to $T$ with the same number of events, and vice versa (insertions and deletions are symmetrical operations, and any substitution can be reversed by a substitution). We thus have ${\rm LRF}(T', T) \leq {\rm LRF}(T, T')$ and ${\rm LRF}(T, T') \leq {\rm LRF}(T', T)$, and equality follows. □

**Proof of Lemma 3**

Proof.
Let ${{\mathcal P}}$ be a path of edge edit operations and label flip transforming $T$ into $T'$ such that $|{{\mathcal P}}|={\rm ELRF}(T,T')$. Then, the sequence ${{\mathcal P}}'$ obtained from ${{\mathcal P}}$ by replacing each edge extension by the corresponding node insertion, each edge contraction by the corresponding node deletion and each node flip by the corresponding node substitution is clearly a path of node edit operations of size $|{{\mathcal P}}'|=|{{\mathcal P}}|={\rm ELRF}(T,T')$ transforming $T$ into $T'$. And thus ${\rm LRF}(T,T') \leq {\rm ELRF}(T,T')$. Figure 2 depicts an example where the inequality is strict. □

**Proof of Lemma 4**

Proof.
For $1 \leq i \leq k$, let $e_i = (x_i, y_i)$, $y_i$ being a leaf of $I$, and let $Y_i = L(T_{y_i})$. As $\cup_i Y_i = \mathcal{L}$, any subtree $I'$ of $T'$ containing the set $\{e'_i\}_{1 \leq i \leq k}$ as $I'$-terminal edges does not contain any other $I'$-terminal edge. As $T'$ is a tree, for any $1 \leq i \neq j \leq k$, there is only one possible path from $x'_i$ to $x'_j$. Uniqueness follows. Suppose that such a subtree $I'$ is not an island. Then, it contains an internal good edge $e' = (x',y')$. In other words, there is a non-trivial bipartition of $\{Y_i\}_{1 \leq i \leq k}$ which is also a bipartition in $I$. This contradicts the fact that $I$ is an island of $T$. Finally, as all terminal edges of $I'$ are good edges of $T'$, it follows that $I'$ is an island of $T'$. □

**Proof of Lemma 5**

Proof.
The scenario depicted above for transforming $I$ into $I'$ clearly requires $\epsilon(I) + \epsilon(I')$ node insertions and deletions, and an additional node label substitution in case $I$ and $I'$ are label-disjoint. We can conclude that ${\rm LRF}(I,I') \leq \epsilon(I) + \epsilon(I')$ if $I$ and $I'$ share a common label and ${\rm LRF}(I,I') \leq \epsilon(I) + \epsilon(I') +1$, if $I$ and $I'$ are label-disjoint. On the other hand, as all the edges of $I$ are bad edges, they should be all removed, before reinserting those of $I'$. Now, since an edit operation can remove or insert at most one edge, and the only operations removing an edge are node removal or node insertion, we clearly require at least $\epsilon(I) + \epsilon(I')$ node removals and insertions to transform the unlabeled form of the tree $I$ into the unlabeled form of $I'$. Furthermore, as deletions do not affect star nodes, at least one node in $I$ should survive (i.e., not be affected by a node deletion). Thus, if the two trees are label-disjoint, then at least one node label substitution is required. We can then conclude that ${\rm LRF}(I,I') \geq \epsilon(I) + \epsilon(I')$ if $I$ and $I'$ share a common label and ${\rm LRF}(I,I') \geq \epsilon(I) + \epsilon(I')+1$, if $I$ and $I'$ are label-disjoint, which concludes the proof. □

**Proof of Lemma 6**

Proof.
Let ${{\mathcal P}}=(o_1, o_2, \cdots o_p)$ be a shortest path transforming $T$ into $T'$. Denote $\epsilon (T_k,T') = \sum_{(I,I') \in {{\mathcal I}}(T_k,T')} \epsilon(I)+\epsilon (I')$, and $\xi(T_{k},T')$ the number of label-disjoints pairs of ${{\mathcal I}}(T_{k},T')$. Assume ${{\mathcal P}}$ contains an operation reducing the number of islands of $T_{i-1}$, and let $o_i$ be the last operation of that form, i.e. $|{{\mathcal I}}(T_{i-1}, T')| > |{{\mathcal I}}(T_{i}, T')|$. Such an operation can only be a deletion $Del(T_{i-1},x,y)$ where $e=\{x,y\}$ is a good edge, thus merging the two islands $I_x$, $I_y$ containing this good edge. As, by assumption, $o_i$ is the last operation merging two islands, at that point each pair of islands is treated separately, and we deduce from the fact that ${{\mathcal P}}$ is a shortest path that ${\rm LRF}(T_{i},T')=\sum_{(I,I') \in {{\mathcal I}}(T_i,T')} {\rm LRF}(I,I')$, and thus

${\rm LRF}(T_{i-1},T')= 1 +\sum_{(I,I') \in {{\mathcal I}}(T_i,T')} {\rm LRF}(I,I')$ . Then, from Lemma 5, ${\rm LRF}(T_{i-1},T')= 1 + \epsilon(T_i,T')+\xi(T_i,T')$. On the other hand, there is a path from $T_{i-1}$ to $T'$ of size $c(T_{i-1},T')=$$\epsilon(T_{i-1}, T') + \xi(T_{i-1},T')$. As $o_i$ is a deletion of a good edge $e=\{x,y\}$, it destroys the bipartition defined by this edge in $T_{i-1}$, consequently the corresponding edge in $T'$ becomes a bad edge. Therefore, $\epsilon(T_{i-1}, T')=\epsilon(T_{i}, T')-1$. On the other hand, let $\delta = \xi(T_{i},T')-\xi(T_{i-1},T')$ be the difference between the number of label-disjoint pairs of islands after performing the operation $o_i$ merging two pairs of islands $(I_1,I'_1)$ and $(I_2, I'_2)$.

- If both pairs $(I_1,I'_1)$ and $(I_2, I'_2)$ share a common label, then the merged pair also shares a common label, and thus $\delta=0$;
- If both pairs $(I_1,I'_1)$ and $(I_2, I'_2)$ are label-disjoint, then after the merging the resulting pair of islands may or may not share a common label and thus $-2 \leq \delta \leq -1$;
- If only one of the two pairs $(I_1,I'_1)$ and $(I_2, I'_2)$ share a common label, then after the merging, the resulting pair of islands may or may not share a common label and thus $-1 \leq \delta \leq 0$.

Therefore, in all cases we have $\xi(T_{i},T') \leq \xi(T_{i-1},T')\leq \xi(T_{i},T') +2$. Recall $c(T_{i-1},T')= \epsilon(T_{i-1}, T') + \xi(T_{i-1},T') = \epsilon(T_{i}, T') -1 + \xi(T_{i-1},T')$. Thus $c(T_{i-1},T') \leq \epsilon(T_{i}, T')-1 + \xi(T_{i},T') +2 = \epsilon(T_{i}, T')+ \xi(T_{i},T')+1$$= {\rm LRF}(T_{i-1}, T')$. As ${\rm LRF}(T_{i-1}, T')$ is the size of the shortest path from $T_{i-1}$ to $T'$, we should have $c(T_{i-1},T') = {\rm LRF}(T_{i-1}, T')$. Therefore, replacing the sequence of operations $(o_i, \ldots o_p)$ on $T_{i-1}$ by a sequence of operations solving each pair of islands separately leads to the same number of operations. □

**Proof of Theorem 1**

Proof.
Let ${{\mathcal P}}$ be a shortest path transforming $T$ into $T'$ verifying the condition of Lemma 6, that is, not involving any removal of good edges. As islands can only share good edges, and good edges are never removed by any operation of ${{\mathcal P}}$, islands are never merged during the process of transforming $T$ into $T'$, and thus can be treated separately. Let ${{\mathcal P}}_i$, $1 \leq i \leq n$, be the subpath of edit operations transforming $I_i$ into $I'_i$. Each ${{\mathcal P}}_i$ should be a shortest path from $I_i$ to $I'_i$ as otherwise it can be replaced by a shortest path, contradicting the fact that ${{\mathcal P}}$ is a shortest path. □

#### Algorithm Implementation

The algorithm $LRF$ (Algorithm 1) consists of four tree traversals, followed by one traversal of a table $X$ of $n$ row, where $n$ is the number of leaves of the trees $T1$ and $T2$. The first tree traversal (Algorithm 2) is essentially Day’s $BUILD(T,X)$ function.

The only noteworthy difference is that each row of $X$ contains additional attributes (i.e., additional columns): a Boolean variable to indicate whether a clade is shared among $T_1$ and $T_2$ (i.e., whether it is a “good” clade), and for good clades, the size and labels of the islands of $T_1$ and $T_2$ rooted in these clades. We also store the clade information in each internal node of $T_1$ (line 17). Like in Day (1985), all the operations performed at each node of the traversal are $\mathcal{O}(1)$, giving an overall runtime of $\mathcal{O}(n)$ for this first traversal.

The second tree traversal (Algorithm 3) is similar to Day’s $COMCLUST$ function. It traverses $T_2$ in postorder, renumbering the leaves in a way consistent with $X$ (line 4), but keeping track not only of the minimum and maximum leaf number contained in each clade but also the number of such leaves. If a clade of $T_2$ is in $T_1$, the set of leaves comprised in that clade will satisfy the property of forming a contiguous sequence $L,L+1,..,R$ of length $R-L+1$ (l. 12). Furthermore, that clade will be stored in $X$ either at row $L$ (line 13) or $R$ (l. 15); either way, we mark that clade as good (l. 14 or 16). For good clades, we store the clade information in each internal node of the tree (l. 15, l. 18); else we store a dummy clade [1,1] (l. 20). Like in Day (1985), all the operations performed at each node of the traversal are $\mathcal{O}(1)$, giving an overall runtime of $\mathcal{O}(n)$ for this second traversal as well.

Finally, the third and fourth tree traversals are performed using $getIslands$ (Algorithm 4). As two islands are separated by a good edge, we can identify an island by the good edge (i.e., the good clade) in which it is rooted, and can thus store island information in the row of $X$ which corresponds to that good edge. We thus traverse the tree, keeping track for each island of (i) the set of labels found in each island (attributes $islandLabels1$ and $islandLabels2$); (ii) the number of bad edges in each island (attributes $islandSize1$ and $islandSize2$). Lines 2–27 define the recursive function used to traverse the tree. We check whether a particular node is attached to the previous island by a good edge or a bad edge (l. 4–9). If it is a good clade (l. 10–17), we have just transitioned to a new island and thus initialize the island label sets and island size (l.13–14 for $T_1$ or l. 16–17 for $T_2$). If it is a bad clade (l. 18–25), then we are still part of previous island, so we just update the set of leaves of the previous island (l. 21 or l. 24), and increment its size counter by one (l. 22 or l. 25). All operations performed at each internal node are constant time, and the number of internal nodes is $\mathcal{O}(n)$, so the time complexity of the tree traversal is done in time $\mathcal{O}(n)$.

Theorem 2. The time complexity of algorithm 1 is $\mathcal{O}(n).$

Proof.
As described above, the algorithm consists of four tree traversal, each running in $\mathcal{O}(n)$, and one traversal of the table $X$ containing $n$ rows, with a constant number of operation per row. This gives an overall time complexity of $\mathcal{O}(n)$. □
